# Increasing Trend of Resistance to Penicillin, Tetracycline, and Fluoroquinolone Resistance in *Neisseria gonorrhoeae* from Pakistan (1992–2009)

**DOI:** 10.1155/2011/960501

**Published:** 2011-09-15

**Authors:** Kauser Jabeen, Summiya Nizamuddin, Seema Irfan, Erum Khan, Faisal Malik, Afia Zafar

**Affiliations:** Department of Pathology and Microbiology, Aga Khan University, Karachi 74800, Pakistan

## Abstract

Emergence and spread of drug resistant Neisseria gonorrhoeae is global concern. We evaluated trends of antimicrobial resistance in Neisseria gonorrhoeae over years 1992–2009 in Pakistan. Resistance rates were compared between years (2007–2009) and (1992–2006). Antimicrobial susceptibility testing was performed and interpreted according to Clinical Laboratory Standards Institute (CLSI) criteria using the disk diffusion methodology against penicillin, ceftriaxone, tetracycline and ofloxacin. Additional antibiotics tested in 100 strains isolated during 2007–2009, included cefotaxime, cefoxitin, cefuroxime, cefipime, ceftazidime, ceftizoxime, cefixime, cefpodoxime, spectinomycin and azithromycin. Neisseria gonorrhoeae ATCC 49226 was used as control. Chi-square for trend analysis was conducted to assess resistance trend over the study period. During study period significant increase in combined resistance to penicillin, tetracycline and ofloxacin was observed (*P* value <0.01). Resistance rates during the two study period also increased significantly (*P* value <0.01). Ceftriaxone resistance was not observed. None of the isolates were found to be resistant or with intermediate sensitivity to additional antibiotics. Our findings suggest that penicillin, ciprofloxacin, tetracycline should not be used in the empirical treatment of gonorrhea in Pakistan. Ceftriaxone and cefixime should be the first line therapy; however periodic MICs should be determined to identify emergence of strains with reduced susceptibility.

## 1. Introduction

Gonorrhea continues to be a public health problem with the emergence of multidrug resistant strains [1]. Accurate diagnosis with effective treatment to prevent further transmission is one of the essential elements for control of gonococcal infections [2]. Treatment strategies should be devised to utilize appropriate and preferably single-dose therapy that could be conveniently administered at the time of diagnosis [3]. Surveillance of *Neisseria gonorrhoeae* antimicrobial resistance is crucial in guiding empirical therapy in any individual setting as resistance may vary in different countries [4]. 

*Neisseria gonorrhoeae* infections have been reported from Pakistan as a cause of sexually transmitted infections, but unfortunately national surveillance data is not available to assess its prevalence or antimicrobial resistance [5, 6]. Due to limited resources and lack of trained technologist and microbiologists, most of the laboratories do not report *Neisseria gonorrhoeae*. Therefore, existing antimicrobial resistance data from the country is limited and mainly laboratory based. Previously high resistance rates to penicillin, tetracycline, and quinolone have already been reported from the country [7, 8]. Thus ongoing surveillance of resistance is important considering the public health importance of gonococcal infections. In this study, we have evaluated antimicrobial resistance in *Neisseria gonorrhoeae* for the years (2007–2009) and compared it with previously reported data (1992–2006). Additionally, 100 strains from 2007–2009 were further analyzed for additional treatment options.

## 2. Materials and Methods

This study was conducted in the clinical laboratory of the Aga Khan University Hospital (AKUH), Karachi, Pakistan. The AKUH laboratory is accredited with the Joint Commission of International Accreditation (JCIA) and routinely participates in the external quality assurance programme of College of American Pathologists (CAP). The laboratory has a well-established national specimen collection network with more than 175 collection units located in major cities and towns across the country. Specimens are requested by physicians as per their judgment and are processed in the clinical laboratory based in Karachi. The laboratory data presented in this study although was not collected through a programmed survey, but represents strains prevalent across the country.

### 2.1. Collection of Isolates

All *Neisseria gonorrhoeae* strains isolated from clinical samples were evaluated for their susceptibility pattern. Information regarding the age, sex, year of isolation, and specimen type was retrieved from the computerized database.

### 2.2. Laboratory Methods

*Neisseria gonorrhoeae* was isolated and identified using standard microbiological procedure [9]. All isolates in 2007–2009 were saved in glycerol phosphate broth and stored at −80°C.

### 2.3. Antimicrobial Susceptibility Testing

During the study period, antimicrobial susceptibility testing was performed and interpreted according to Clinical Laboratory Standards Institute (CLSI) criteria using the disk diffusion methodology [10]. All isolates were tested against penicillin (10 *μ*g), ceftriaxone (30 *μ*g), tetracycline (30 *μ*g), and ofloxacin (5 *μ*g) (Oxoid) on GC agar base and 1% defined growth supplement. Minimum inhibitory concentration (MIC) of ofloxacin was determined in strains isolated during 2007–2009 by the E-test method (AB biodisk, Sweden) on GC agar base and 1% defined growth supplement. MIC with E test was performed as specified by the manufacturer. Additional antibiotics tested in selected strains isolated during 2007–2009 included cefotaxime (30 *μ*g), cefoxitin (30 *μ*g), cefuroxime (30 *μ*g), cefipime (30 *μ*g), ceftazidime (30 *μ*g), ceftizoxime (30 *μ*g), cefixime (30 *μ*g), cefpodoxime (30 *μ*g) spectinomycin (100 *μ*g), and azithromycin (15 *μ*g) (Oxoid). The antimicrobial susceptibility of all these antibiotics was interpreted according to CLSI criteria except azithromycin that was interpreted using British Society of Antimicrobial Chemotherapy (BSAC) methods for antimicrobial susceptibility testing [11]. Neisseria* gonorrhoeae* ATCC 49226 was used as control for disc diffusion and MIC testing. Beta-lactamase production was checked using nitrocefin reagent. Strains resistant to penicillin, tetracycline, and ofloxacin were categorized as having combined resistance to penicillin, tetracycline, and ofloxacin. 

### 2.4. Data Management and Statistical Analysis

Data extracted from the computerized information system were transferred to the statistical software SPSS version 14.0. Frequencies with percentages were computed for each year. Chi-square for trend analysis was also conducted to assess resistance trend over the study period. A *P*-value of less than 0.5 was considered statistically significant.

## 3. Results

A total of 804 *Neisseria gonorrhoeae* strains were isolated and identified during 1992–2009. Of which 82% isolates were from males and 18% were from females. Majority (93%) of organisms were isolated from urethral, high vaginal, and cervical swabs, (6%) were from pus aspirates, and (1%) were from eye swabs, blood, and urine.

**Figure 1:**
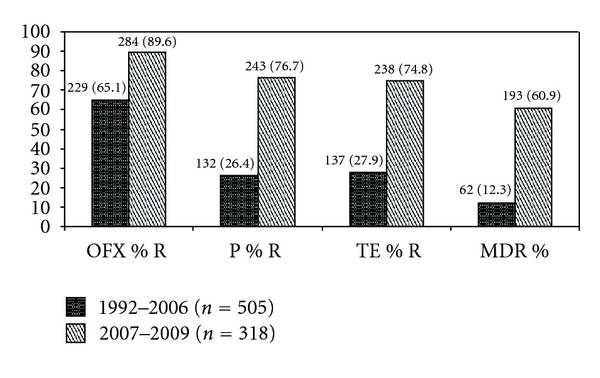
Comparison of resistance in *Neisseria gonorrhoeae *strains during years 1992–2006 and 2007–2009. There is a significant increase (*P*-value <0.01) in resistance to penicillin, tetracycline, ofloxacin, and combined resistance to all 3 antibiotics over these two periods. n is the total number of strains for that particular period.

During the study period, an increasing trend of resistance was observed against penicillin, tetracycline, and ofloxacin (*P*-value for trends <0.01) (Table 1). *N. gonorrhoeae* strains with combined resistance to penicillin, tetracycline, and ofloxacin increased from (0%) in 1992 to (70.8%) in 2009 (*P*-value for trends <0.01).  Table 2 shows *N. gonorrhoeae* strains showing intermediate susceptibility during the study period. No resistant strains to ceftriaxone were picked up by the disk diffusion method during the 15-year period. Comparison of resistance rates during years 1992–2006 and 2007–2009 revealed significantly increased resistance between the two periods (Figure 1).

Susceptibility to cefotaxime, cefoxitin, cefuroxime, cefipime, ceftazidime, ceftizoxime, cefixime, cefpodoxime, spectinomycin, and azithromycin was determined in 100 isolates. Resistance against the above-mentioned agents was not identified, and 100% of the tested isolates were found susceptible.

## 4. Discussion

The results of our study demonstrated increasing trend of resistance in* N. gonorrhoeae *against penicillin, tetracycline, and ofloxacin in Pakistan. The resistance rates reported in this study against first-line drugs markedly exceed the World Health Organization cutoff of 5%, precluding their use for the empirical therapy in gonococcal infections [12]. Accurate information on antimicrobial susceptibility of prevalent *N. gonorrhoeae *is, thus, essential for the treatment and control of this disease. Keeping in view the current scenario in Pakistan and absence of a national guideline for treatment of gonococcal infections, the selection of appropriate antibiotics for empirical treatment of gonorrhea is challenging.

This 10-year analysis clearly showed that the level of resistance to traditional antibiotics used in the treatment of gonorrhea continued to increase in local isolates. This trend follows the global pattern of antibiotic resistance in *N. gonorrhoeae *and leaves third-generation cephalosporins as the recommended antibiotic for its treatment [13]. However emergence and spread of strains with reduced susceptibility to ceftriaxone is another emerging issue. Unfortunately, these strains are simultaneously resistance to multiple drug classes, including quinolones, macrolides, penicillins, and tetracyclines [14]. We did not perform ceftriaxone MIC, required to detect reduced susceptibility, and there is a possibility to miss their occurrence as the frequency of MDR strains constitutes a large proportion of our current *N. gonorrhoeae* isolates circulating in the community. On the other hand, treatment failure with ceftriaxone has not been documented from the country. The best strategy in this scenario would be periodic determination of ceftriaxone MICs to detect existence of these strains and close followup with clinicians to identify treatment failure.

 A high quinolone resistance approaching almost 100% in recent years was demonstrated in local isolates. This exponential rise in quinolone resistance has also been reported from the region and raises several concerns [15, 16]. We had not evaluated the exact reason for this finding, but probable reasons could be its continuous use despite higher rate of resistance and poor disease control in the community. Increased resistance rates to quinolones have been reported from the country in other community acquired pathogens highlighting the overall misuse of quinolones as the major reason of increased resistance [17, 18].

In view of high resistance to the currently tested antimicrobials in our setting, we tested 8 cephalosporins including 3 oral options: cefixime, cefpodoxime, and cefuroxime. The efficacy of cefixime has been shown to be similar to ceftriaxone [19]. Although cefpodoxime and cefuroxime have shown a cure rate of more than 95%, their pharmacodynamic parameters are less favorable than those of cefixime and ceftriaxone [20, 21]. Nevertheless, they could be used as oral alternatives in the treatment of uncomplicated gonorrhea in our setting [22]. Cefotaxime, cefoxitin, cefepime, ceftazidime, and ceftizoxime are the other single-dose cephalosporins included in the updated treatment regimens for gonococcal infections by the Centers for Disease Control and Prevention [23]. Although studies have reported emergence of resistance to these agents, we were not able to detect resistance against any of them in this study.

Local isolates were also uniformly susceptible to spectinomycin, which is a good option especially in those patients who cannot tolerate cephalosporins [24]. The most likely reason for its consistent susceptibility is its less-frequent use in the community. Rapid emergence of resistance has been reported with the wider use of spectinomycin mandating its rationale use. 

Though azithromycin is so far not recommended as a single agent for the treatment of gonorrhea, it has a potential role in the management of these infections in combination with third-generation cephalosporin. We did not detect resistance to this drug although strains with high level resistance have emerged as another threat [25, 26].

Although our dataset included samples from all over Pakistan, sampling limitations prevent us from generalizing our results to the entire population of the country. However, it is important to note that only a few laboratories in the country perform antimicrobial susceptibility testing in *Neisseria gonorrhoeae*, and our data is the largest ever from the country reporting resistance over time. Another limitation is lack of MICs determination for cephalosporins with possibility of missing strains with reduced susceptibility.

In conclusion, we recommend that penicillin, ciprofloxacin, and tetracycline should not be used in the empirical treatment of gonorrhoeae. Ceftriaxone and cefixime should be the first line therapy; however, periodic MICs should be determined to identify emergence of strains with reduced susceptibility. Optimized, standardized, and quality assured antibiotic susceptibility testing needs to be established in laboratories in Pakistan. Simultaneously prevention strategies should be strengthened to maximize the clinical utility of these antimicrobials. Such strategies could include the use of a limited number of treatment regimens and overall avoiding a general misuse of the remaining yet effective antibiotics. In addition, a national policy for management of gonococcal infections should be developed and disseminated to clinicians in the country.

## Figures and Tables

**Table 1:** Antimicrobial resistance trends in *Neisseria gonorrhoeae* strains over the years 1992–2009 showing significant increase in resistance to penicillin, tetracycline, and ofloxacin. There is also significant increase in combined resistance to all 3 antibiotics. *n* is the total number of resistance strains for that particular year. Percentage is shown in parenthesis (%).

Years	Total number of strains	CRO	OFX	P	TE	MDR
		*n* (%)	*n* (%)	*n* (%)	*n* (%)	*n* (%)
1992	15	0	0	1 (7.7)	0	0
1993	11	0	0	0	0	0
1994	16	0	0	4 (25)	0	0
1995	28	0	0	1 (3.6)	0	0
1996	20	0	0	0	0	0
1997	25	0	0	8 (32)	5 (20)	0
1998	26	0	0	3 (11.5)	1 (3.8)	0
1999	33	0	6 (28.8)	6 (19.4)	3 (9.7)	0
2000	25	0	6 (24)	2 (8)	0	0
2001	26	0	6 (23.1)	5 (19.2)	1 (3.8)	0
2002	19	0	8 (42.1)	1 (5.3)	2 (10.5)	0
2003	51	0	37 (72.5)	15 (29.4)	13 (25.5)	7 (13.7)
2004	56	0	47 (83.9)	29 (51.8)	26 (46.4)	17 (30.4)
2005	61	0	48 (78.7)	18 (29.5)	36 (59)	13 (21.3)
2006	90	0	71 (78.9)	39 (43.3)	49 (54.4)	25 (27.8)
2007	95	0	79 (83.2)	57 (60)	62 (65.3)	39 (41.1)
2008	116	0	107 (92.2)	94 (81)	93 (80.2)	79 (68.1)
2009	106	0	98 (92.5)	92 (86.8)	82 (77.6)	75 (70.8)
*P*-value for trends		NA	<0.01	<0.01	<0.01	<0.01

P: Penicillin; TE: Tetracycline; OFX: Ofloxacin; CRO: Ceftriaxone; MDR: strains resistant to penicillin, tetracycline, and ofloxacin.

**Table 2:** *Neisseria gonorrhoeae* strains with intermediate susceptibility over the study period 1992–2009. *n* is the total number of intermediate strains for that particular year. Percentage is shown in parenthesis (%).

Years	*n*	OFX	P	TE
1992	15	0	1 (7.7)	0
1993	11	0	1 (9.1)	0
1994	16	0	5 (31.3)	3 (23.1)
1995	28	0	9 (32.1)	8 (28.6)
1996	20	0	1 (5.3)	2 (10)
1997	25	0	7 (28)	7 (28)
1998	26	0	3 (11.5)	4 (15.4)
1999	33	1 (4.8)	3 (9.7)	8 (25.8)
2000	25	0 (0)	4 (16)	3 (12)
2001	26	2 (7.7)	2 (7.7)	3 (11.5)
2002	19	1 (5.3)	10 (52.6)	2 (10.5)
2003	51	5 (9.8)	22 (43.1)	6 (11.8)
2004	56	0 (0)	13 (23.2)	3 (5.4)
2005	61	2 (3.3)	36 (59)	5 (8.2)
2006	90	9 (10.1)	42 (46.7)	15 (16.7)
2007	95	4 (4.2)	31 (32.6)	23 (24.2)
2008	116	5 (4.3)	16 (13.8)	17 (14.7)
2009	106	6 (5.7)	12 (11.3)	16 (15.1)

P: Penicillin; TE: Tetracycline; OFX: Ofloxacin.
